# Prognostic value of the NLR combined with CIP2A in the serum of patients with colorectal cancer

**DOI:** 10.1186/s12893-021-01273-5

**Published:** 2021-06-18

**Authors:** Wei Chen, Hong-Jun Yi, Xiao-Qiong Chen, Wan-Zhen Xie, Xing-kui Tang, Jun-Wen Ye, Xiang Peng, Yan Zhang, Jing-Lin Liang, Mei-Jin Huang

**Affiliations:** 1 Department of Colorectal Surgery, The Sixth Affiliated Hospital of Sun Yat-Sen University, 510655 Guangzhou, People’s Republic of China; 2 Guangdong Provincial Key Laboratory of Colorectal and Pelvic Floor Disease, The Sixth Affiliated Hospital of Sun Yat-Sen University, Guangzhou, 510655 China; 3 Guangdong Research Institute of Gastroenterology, The Sixth Affiliated Hospital of Sun Yat-Sen University, Guangzhou, 510655 China; 4 Department of Pathology, The Sixth Affiliated Hospital of Sun Yat-Sen University, #26 Yuancun Erheng Road, Guangzhou, 510655 Guangdong China; 5 School of Humanities and Social Sciences, Zhongkai University of Agriculture and Engineering, Guangzhou, 510225 China; 6 Guangzhou Panyu Central Hospital, Guangzhou, 511400 Guangdong People’s Republic of China; 7 Department of Gastroenterology, The Sixth Affiliated Hospital of Sun Yat-Sen University, Guangzhou, 510655 China; 8 Department of Medicine Oncology, The Sixth Affiliated Hospital of Sun Yat-Sen University, Guangzhou, 510655 China

**Keywords:** CIP2A, NLR, Colorectal cancer, Serum, Prognosis

## Abstract

**Objective:**

This study aimed to investigate the prognostic value of CIP2A (cancerous inhibitor of protein phosphatase 2A) and the NLR (neutrophil–lymphocyte ratio) in the serum of patients with CRC (colorectal cancer) after resection.

**Methods:**

The clinicopathological data of 61 patients who underwent resection between January 2012 and December 2013 were collected. The NLR and CIP2A were divided into low score groups (0) and high score groups (1) with 2.03 and 6.07 as the optimal cut-off value according to the receiver operating characteristic (ROC) curve analysis. To identify the COCN (combination of CIP2A and the NLR) score, we added CIP2A and NLR points together and categorized CRC patients into three groups. Kaplan–Meier curves were used to identify the overall survival (OS) rates of the different groups. Finally, a ROC curve was plotted to evaluate the prognostic efficacy of COCN.

**Results:**

The CIP2A was associated with location (P = 0.046) and CEA (P = 0.037) in patients with CRC. Kaplan–Meier survival curves showed that the 5-year OS of patients with low level of serum CIP2A was better than that of high level. The 5-year OS of the patients in the low NLR group was better than that of those in the high NLR group. The COCN score was associated with CEA (P < 0.001) and CA19-9 (P = 0.001). The 5-year OS of the patients in the COCN 0 group was highest, followed by that of those in the COCN 1 and COCN 2 groups. Age, N stage and M stage were factors associated with 5-year OS according to the univariate and multivariate analyses (P < 0.05). The area under the curve (AUC) for COCN was largest, indicating that COCN has better prognostic power than CIP2A or the NLR alone.

**Conclusion:**

COCN could be used as a better prognostic biomarker for CRC than the NLR or CIP2A alone.

## Background

In recent years, the incidence of colorectal cancer (CRC) has risen rapidly [1]. Despite the increased prevalence of chemoradiotherapy along with surgery, the overall survival (OS) rate is still not satisfactory [2]. Therefore, there are urgent that needs to find reliable indicators of the prognosis and improve treatment strategies.

Recently, translational research associated with CRC has identified a wide spectrum of potential biomarkers that could be used for clinical diagnosis, treatment, and follow-up, such as imaging, circulating biomarkers and eliminated metabolites [3]. Inflammation is one of the main factors affecting the condition of CRC patients. Because their inflammation can be represented relatively well by the neutrophil–lymphocyte ratio (NLR), timely preoperative intervention can provide benefits to CRC patients. The NLR, which is a systemic inflammatory response (SIR) marker has been shown to be associated with the prognosis in patients with various types of cancer [4–7]. In addition, the survival of patients is associated not only with the host SIR but also with tumour characteristics [8]. In addition, the cancerous inhibitor of protein phosphatase 2A (CIP2A) is a newly recognized oncoprotein that plays a key roles in maintaining cell phenotype, promoting cell proliferation and forming tumours [9, 10]. CIP2A has been found to be overexpressed in the serum and cells of patients with different types of cancers, such as hepatocellular carcinoma (HCC), prostate carcinoma and breast carcinoma [11–14]. To date, the value of CIP2A in the serum of patients combined with the NLR for revealing prognosis has not been reported. We hypothesized that identifying parameters reflecting both tumour characteristics and the host SIR may be a good approach for reflecting patient survival and that COCN (combination of CIP2A and NLR) may be a good biomarker for the prognostic assessment of CRC.

This knowledge was the impetus for this study, which aimed to explore the clinical value of the NLR combined with CIP2A in the serum of patients undergoing resection for prognosis by analysing the postoperative relationship of COCN with CRC.

## Materials and methods

### Patients

**Fig. 1:**
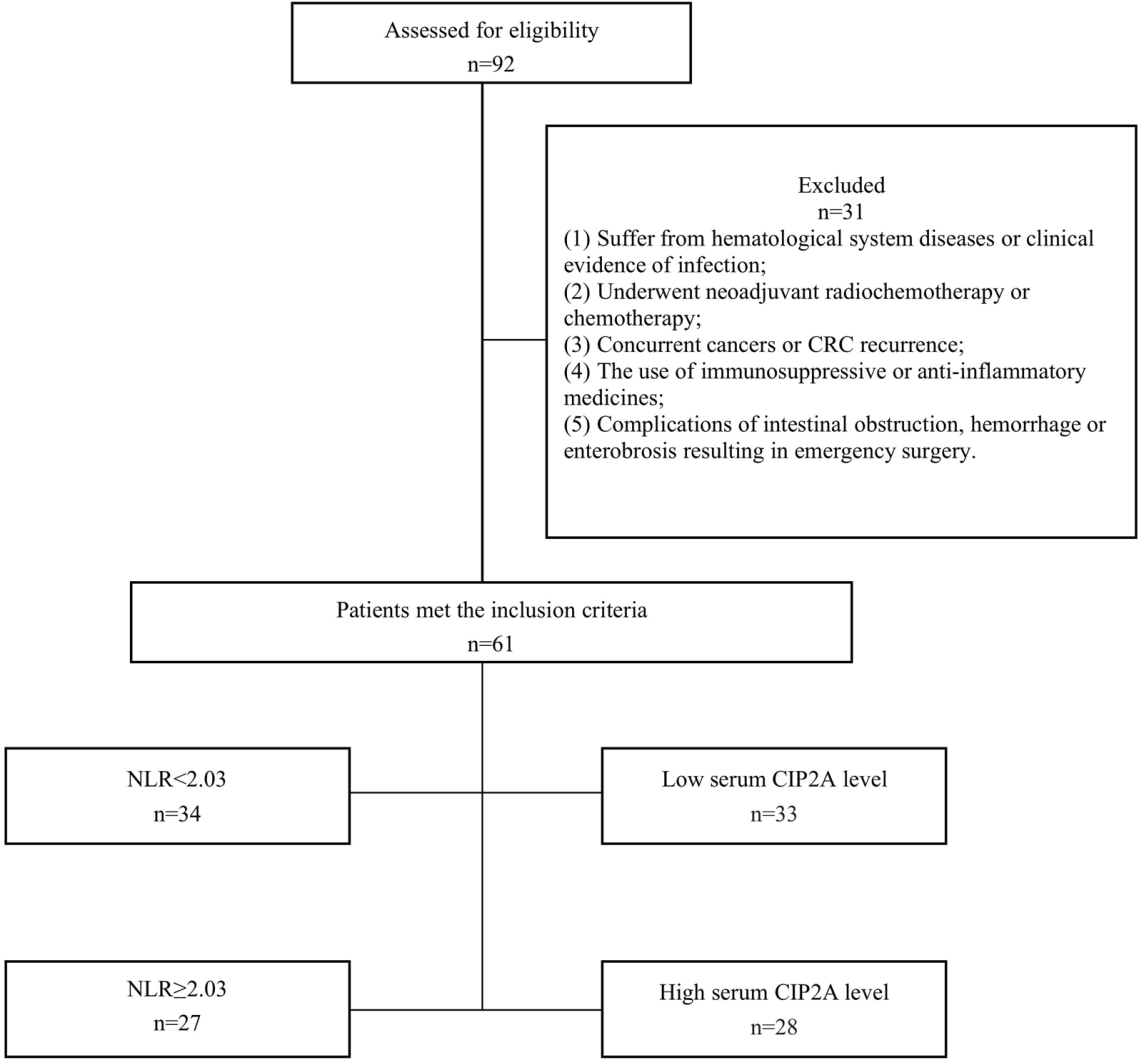
The flow of patients through the study

This retrospective analysis included data from the hospital records of 92 consecutive patients who underwent surgery for colorectal cancer at The Sixth Affiliated Hospital of Sun Yat-sen University between January 2012 and December 2013. The flow of patients through the study is visualised in Fig. 1.

The inclusion criteria for patient enrollment were shown as follows: (1) postoperative pathology confirmed CRC; (2) patients who underwent radical surgery; and (3) the availability of complete peripheral blood counts and follow-up data. The exclusion criteria were shown as follows: (1) suffer from hematological system diseases or clinical evidence of infection; (2) underwent neoadjuvant radiochemotherapy or chemotherapy; (3) concurrent cancers or CRC recurrence; (4) the use of immunosuppressive or anti-inflammatory medicines. and (5) complications of intestinal obstruction, hemorrhage or enterobrosis resulting in emergency surgery. Finally, 61 cases were enrolled in the present study. Disease-free survival (DFS) and overall survival (OS) are the primary study endpoints. At the end of the follow-up period (2020), the median follow-up is 207 months (range, 17–260 months).

### Data collection

The CEA (carcinoembryonic antigen), CA199 (carbohydrate antigen 199), the neutrophil count, the lymphocyte count, the red blood cell (RBC) count, and the platelet count were evaluated within 3 days before the surgery. NLR = neutrophil rate (%)/lymphocyte rate (%). All methods were carried out in accordance with relevant guidelines and regulations.The pathological stage was established in accordance with the eighth edition of AJCC/IUCC Staging System.

Patients were followed up regularly every year. Routine examination included chest and abdominal CT scans, tumour markers evaluations, and colonoscopy. Written informed consent was obtained from all subjects.The study was approved by the Institute Research Medical Ethics Committee of Sun Yat-Sen University.

### Elisa

5 ml of peripheral blood samples were collected and put into the separating gel vacuum tubes (try to complete the separation of serum in 2 h). After standing for 1 h at room temperature, the procoagulant blood was centrifugated at 2500r/min for 10 min, then carefully extracted the upper clear liquid (serum) with a pipettor, and packed into two sterilized EP tubes according to 1 ml / tube. Finally the serum is stored in − 80℃ freezer in 3 h.

The CIP2A ELISA assays (https://cdn.mybiosource.com/tds/protocol_manuals/800000-9999999/MBS2020013.pdf) was performed according to the manufacturers' instructions. After the experiment was stopped, the absorbance at 490 nm was identified. The obtained values were used to determine the serum CIP2A level of the tested individuals based on the standard curve.

### Statistical analysis

Either the Wilcoxon rank-sum test or independent sample t-test was used to analyze the continuous variables, while the categorical variables were analyzed using either the Fisher’s exact test or Pearson’s chi-squared test where applicable. The receiver operating characteristic (ROC) curve analysis was used to identify the optimal cut-off values of NLR, CIP2A according to the Youden index (maximum = sensitivity + specificity − 1). Any potentially relevant factors derived from the univariate analysis were assessed in the multivariate model using Cox’s regression. The hazard ratios (HR) and 95% confidence intervals (CI) were also calculated. The OS rate was determined by the Kaplan–Meier method, and the log-rank test was used to identify if the result was statistically significant. All statistical analyses were performed by the SPSS 11.0 software and P < 0.05 were identified to be statistically significant.

## Results

### ROC curves for the NLR and its correlation with prognosis in CRC patients

**Fig. 2:**
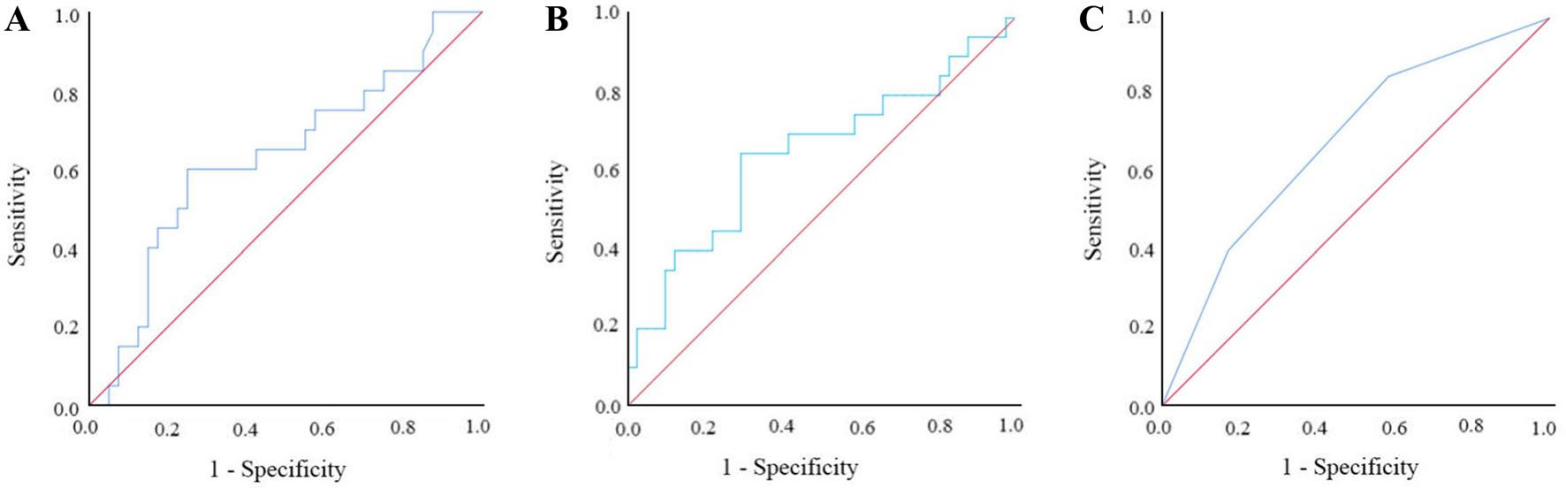
Receiver operating characteristics curve analysis of CIP2A, NLR and COCN in CRC patients. **A** ROC curve analysis of CIP2A for OS. **B** ROC curve analysis of NLR for OS. **C** ROC curve analysis of COCN for OS

ROC curve analysis was used to show the relationship between the NLR and OS (Fig. 2). With the maximum Youden index as the cut-off point, we obtained a cut-off value of 2.03 for the NLR. The corresponding sensitivity was 0.650, the specificity was 0.341, and the area under the curve (AUC) was 0.650 (95%CI: 0.493–0.807). Accordingly, we divided the patients into two groups (a high NLR group ≥ 2.03 and low NLR group < 2.03).

After comparing the clinical data of the patients in the two groups, there were no statistically significant differences in sex, age, CEA, T stage, N stage or CA19-9 (Table 1). The survival rate of the low NLR group was significantly higher than that of the high NLR group (P < 0.05) (Table 2).

**Table 1:** Analysis of NLR, CIP2A level and related factors

Related factor	N	CIP2A		*P*	NLR		*P*
		Low	High		< 2.03	≧ 2.03	
*Gender*				0.052			0.509
Female	30	20	10		18	12	
Male	31	13	18		16	15	
*Age*				0.052			0.509
≥ 65	31	13	18		16	15	
< 65	30	20	10		18	12	
*Location*				0.046			0.062
Colon	33	14	19		22	11	
Rectum	28	19	9		12	16	
*T stage*				0.660			0.405
T1–T2	33	17	16		20	13	
T3–T4	28	16	12		14	14	
*N stage*				0.339			0.149
N0	42	21	21		26	16	
N1–N2	19	12	7		8	11	
*M stage*				0.076			0.265
M0	53	31	22		31	22	
M1	8	2	6		3	5	
*Intestinal obstruction*				0.766			0.682
No	51	28	23		27	24	
Yes	10	6	4		6	4	
*CEA*				0.037			0.990
≥ 5	52	31	21		29	23	
< 5	9	2	7		5	4	
*CA19-9*				0.115			0.268
≥ 37	47	28	19		28	19	
< 37	14	5	9		6	8

**Table 2:** Univariate and multivariate analyses of prognostic factors for 5-year OS in patients with CRC

	Univariable Cox regression			Multivariate Cox regression		
	HR	CL (95%)	*P*	HR	CL (95%)	*P*
*Gender*			0.068			NS
Female	1					
Male	0.411	0.158–1.070				
*Age*			0.005			0.031
< 65	1			1		
≥ 65	3.686	1.468–9.251		3.089	1.111–8.585	
*Location*			0.616			NS
Colon	1					
Rectum	1.296	0.470–3.573				
*T stage*			0.012			0.343
T1–T2	1			1		
T3–T4	1.703	1.122–2.585		1.731	0.556–5.390	
*N stage*			0.013			0.009
N0	1			1		
N1–N2	3.274	1.282–8.359		4.401	1.456–13.304	
*M stage*			0.001			0.010
M0	1			1		
M1	6.016	2.398–15.094		4.575	1.441–14.522	
*Intestinal obstruction*			0.005			0.123
No	1			1		
Yes	3.686	1.468–9.251		2.668	0.766–9.294	
*CEA*			0.125			NS
< 5	1					
≥ 5	2.211	0.802–6.095				
*CA19-9*			0.616			NS
< 37	1					
≥ 37	1.706	0.569–5.111				
*CIP2A*			0.192			NS
Low	1					
High	1.816	0.740–4.456				
*NLR*			0.012			0.519
< 2.03	1			1		
≥ 2.03	3.030	1.206–7.612		1.414	0.493–4.051	
*COCN*			0.020			0.232
0	1			1		
1 + 2	4.829	1.278–18.251		2.714	0.528–13.946	

### ROC curves for CIP2A and its correlation with prognosis in CRC patients

As is shown in Fig. 2, the relationship between the CIP2A and OS was determined by ROC curve analysis. With the maximum Youden index as the cut-off point, we obtained a cut-off value of 6.07 for CIP2A. The corresponding sensitivity was 0.600, the specificity was 0.400, and the area under the curve (AUC) was 0.629 (95%CI: 0.475–0.783). Therefore, we divided the patients into two groups (a high CIP2A group ≥ 6.07 and low CIP2A group < 6.07).

In addition, the level of serum CIP2A was significantly associated with location (P = 0.046) and CEA (P = 0.037). No significant correlations were found between the level of serum CIP2A and some factors, such as the age, sex, intestinal obstruction, T stage, CA19-9 and N stage (Table 1). The survival rate of the low CIP2A group was higher than that of the high CIP2A group. The CIP2A was not associated with OS according to the univariate and multivariate analyses (Table 2).

### Correlations of COCN with clinicopathological factor

**Fig. 3:**
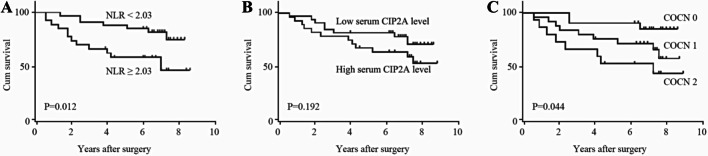
Kaplan–Meier survival curves for OS in 61 patients undergoing primary CRC resection according to their NLR and CIP2A levels. **A** OS according to NLR. **B** OS according to CIP2A. **C** OS according to COCN

As is shown in Table 3, patients were assigned into three groups according to their COCN score. The COCN score was significantly correlated with CIP2A, the NLR, CEA and CA199, while there was no significant correlations observed between COCN and other factors (Table 4). There were a significant differences in the survival rate among the three groups (P < 0.05). As shown in Fig. 3, the survival rate of patients in the COCN 0 group was highest, followed by that of those in the COCN 1 and COCN 2 groups. The 5-year OS rates of the three groups were 90.4%, 71.7% and 53.3%, respectively.

**Table 3:** Prognostic scores of NLR, CIP2A and COCN

Scoring system	Score
*NLR*	
≥ 2.03	1
< 2.03	0
*CIP2A*	
High	1
Low	0
*Combination of CIP2A and NLR(COCN)*	
NLR ≥ 2.03 and CIP2A ( +)	2
NLR ≥ 2.03 or CIP2A ( +)	1
Neither NLR ≥ 2.03 nor CIP2A ( +)	0

*NLR* neutrophil-to-lymphocyte ratio, *CEA* carcinoembryonic antigen

**Table 4:** Relationship between COCN and clinicopathological features of patients with CRC

	COCN				
Variable	2	1		0	*P*
*Gender*					0.237
Female	5	12		13	
Male	10	13		8	
*Age*					0.914
< 65	7	15		16	
≥ 65	8	10		5	
*Location*					0.914
Colon	5	10		8	
Rectum	10	15		13	
*T stage*					0.939
T1–T2	8	13		12	
T3–T4	7	12		9	
*N stage*					0.454
N0	11	15		16	
N1–N2	4	10		5	
*M stage*					0.089
M0	12	20		21	
M1	3	5		0	
*Intestinal obstruction*					0.859
No	13	20		17	
Yes	2	5		4	
*CEA*					< 0.001
≥ 5	3	20		20	
< 5	12	5		1	
*CA19-9*					0.001
≥ 37	5	18		19	
< 37	10	7		2	
*CIP2A*					< 0.001
Low	0	12		21	
High	15	13		0	
*NLR*					< 0.001
< 2.03	0	13		21	
≧ 2.03	15	12		0

We drew ROC curves for the survival rate estimated by CIP2A, the NLR, or COCN to reveal the prognosis of CRC patients to compare the postoperative prognostic values of the three factors. As is shown in Fig. 2, the AUCs of COCN, CIP2A and the NLR were 0.677, 0.629, and 0.650, respectively, indicating that COCN had a better prognostic effect.

## Discussion

As a common malignant tumour worldwide, CRC does not have a good prognosis because of its highly malignant nature and strong heterogeneity [15]. To improve the survival rate, we have worked to improve early detection and early intervention, and studied more about the related factors that affect long-term prognosis after CRC resection in depth.

The SIR is closely related to the CRC patient prognosis [16, 17]. As a potential dynamic balance index, the NLR can reflect inflammation in the body relatively well. It has been proven to be an important prognostic factor for patients with CRC [18, 19]. In addition, CIP2A has a good prognostic effect according to our previous reports [20, 21]. However, each marker has its limitations, and the value of CIP2A combined with the NLR in revealing CRC patient prognosis has not been studied previously. Therefore, this study aimed to explore the preoperative effects of CIP2A, the NLR, COCN and other related factors on the postoperative prognosis of CRC patients.

As a defence response, inflammation plays an important roles in the occurrence and development of tumours. Chronic inflammation can promote tumours, and the inflammation induced by tumours can produce a "snowball" effect, which leads to the continued development of tumours [22]. The NLR ratio reflects the dynamic balance between the body's inflammatory response and antitumour immunity. Ying et al. found that the preoperative NLR is an independent risk factor for OS and DFS after surgery [23]. In the determination of the NLR cut-off value, previous studies have fluctuated over a wide range, from 1.505 to 5.0. Most studies chose 2.81 as the NLR cut-off value [24–27]. In this study, we drew an ROC curve with a cut-off value of 2.03, which is very close to the commonly used NLR cut-off value of 2.81. By analysing the clinical data, no significant correlations were found between NLR and other factors, such as the sex, age, location, T stage, and N stage. This was not consistent with the previous meta-analysis results [28]. Moreover, the univariate analysis showed that the NLR was related to the postoperative prognosis, but the NLR was not an independent risk factor according to the results. Therefore, future studies should involve a greater number of serum samples to identify whether the NLR can be used as a marker.

Postoperative prognosis is related to multiple factors, and a single indicator cannot reflect prognosis. Therefore, the combination of CIP2A and the NLR was used, and their clinical value for the prognosis was evaluated. By analysing the clinical data of CRC patients and COCN, the results showed that higher results for these indicators indicate worse tumour biological behaviours and worse body conditions in patients with CRC, suggesting that the high COCN group might have a worse prognosis, which was consistent with the conclusions obtained from the analysis of CIP2A and the NLR. In addition, we used Kaplan–Meier survival curve analysis to compare the survival rate among three groups, and Cox analysis showed that COCN was not an independent risk factor for OS in CRC patients. The COCN score had the largest AUC, indicating that COCN works better for the reflection of postoperative prognosis than CIP2A or the NLR.

The NLR is an indicator that dynamically balances the body's inflammation and immunity [29]. It also reflects the preoperative inflammation and immune status of CRC patients. Therefore, the combination of CIP2A and the NLR can reflect non-tumour factors, such as the inflammation, immune status, and nutritional status of the body, which can provide a more comprehensive assessment of the preoperative condition of CRC patients. Therefore, this combination has a better prognostic value for the CRC patients. In addition, COCN has a better prognostic value than CIP2A or the NLR. The prognosis of CRC patients in the high COCN group is worst, therefore, for patients in the of COCN 2 group, we should make appropriate preoperative interventions to improve their long-term prognosis.

The study has several limitations. (1) the sample size of patients selected was limited; (2) the study was retrospectively analyzed and prospective multicenter clinical trials are required to further identify these findings.

## Conclusion

In summary, the COCN reveals the long-term prognosis of patients with CRC better than using CIP2A or the NLR alone, which indicates that it is effective and feasible to combine multiple risk factors for postoperative prognosis assessment of patients with CRC, and more combinations can be explored in the future.

## Data Availability

All data generated or analyzed during this study are included in this published article.

## Abbreviations

CIP2A: Cancerous inhibitor of protein phosphatase 2A
CRC: Colorectal cancer
NLR: Neutrophil–lymphocyte ratio
COCN: Combination of CIP2A and NLR
OS: Overall survival
DFS: Disease-free survival
